# Promising Targets and Current Clinical Trials in Metastatic Squamous Cell Lung Cancer

**DOI:** 10.3389/fonc.2014.00320

**Published:** 2014-12-09

**Authors:** Mark D. Vincent

**Affiliations:** ^1^London Regional Cancer Program, Department of Medical Oncology, London Health Sciences Centre, London, ON, Canada

**Keywords:** squamous, epidermoid, lung cancer, systemic treatment, molecular

## Abstract

Squamous cancer of the lung (SQCC), although no longer the premier variant of non-small cell lung cancer, continues to impose a heavy world-wide burden. Advanced SQCC has enjoyed little of the recent progress benefiting patients with adenocarcinoma of the lung, but that has now begun to change. This article reviews the underlying molecular pathology of SQCC, as well as potential new targets and the corresponding novel targeted agents; included are some of which may soon be approvable in this notoriously hard-to-treat indication.

## Introduction

Although squamous (epidermoid) lung cancer (SQCC) represents a declining proportion of non-small cell lung cancer (NSCLC), it still represents about 30% of all NSCLC, and as such, accounted for 6300 of the ±24,700 new cases of lung cancer in Canada thought to have occurred in 2013 (1). There is little if any comprehensive data on the histologic subtype distribution by stage, but it is possible that SQCC is somewhat more frequent in earlier stages as evidenced by two large Canadian series of stage III NSCLC, in both of which SQCC was the most frequent histological subtype (2, 3).

Up to, and including the 1990s, histological subtype was not considered to be particularly relevant in determining either the choice of therapy, or its outcomes, in advanced NSCLC. Of course, it had long been realized that SQCC had certain characteristic clinical features, such as a much higher incidence of hypertrophic pulmonary osteoarthropathy (including “clubbing”), non-metastatic paraneoplastic hypercalcemia and proximally situated, cavitating primary lesions, compared to other types of lung cancer. Furthermore, it had also been well understood that SQCC had a stronger association with smoking than adenocarcinoma (ADC), e.g., for current smokers (RR 16.91 vs. 4.21) (4). Unsurprisingly, SQCC is the histological subtype most associated with emphysema (5).

All of these features, while of great interests to diagnostic physicians and respirologists, may also impact directly or indirectly on the management of advanced NSCLC by oncologists. However, shortly after the turn of the century, it became clear that the histological subtyping of lung cancer had a previously unrecognized importance that went way beyond the fine-tuning of management, and even beyond the important distinction between small cell (SCLC) and NSCLC, which had, heretofore, been the major contribution of pathologists. Two types of new molecularly targeted drugs, gefitinib and bevacizumab, and one new chemotherapeutic, pemetrexed, seemed to have dramatically different effects (either with respect to efficacy or toxicity) according to histology, and the increasingly powerful techniques of genetic sequencing and analysis were revealing that SQCC seemed to be a different molecular entity from other types of lung cancer. In an era in which molecular diagnostics is seen increasingly as a way not only to guide the use of existing therapies but also to select patients for clinical trial accrual, and most critically, as a pathway for novel drug design, the traditional “one size fits all” categorization of advanced NSCLC is increasingly seen as obsolete.

That having been said, it is worth noting that often, definitive biopsy material may not be available, not even for histology let alone molecular tests, and the clinician may be forced to rely on a scant, non-specific cytology specimen (“NSCLC-NOS”), and the clinical features may be the only clue to the true histology. Furthermore, novel immunomodulatory drugs are definitely active in both SQCC and ADC, and for these agents, emerging molecular biomarkers may prove to be more predictive such that histological subtyping of NSCLC may, at least in the immunological arena, again become irrelevant.

## Conventional Pathology

Because histological subtype now profoundly affects clinical management, and because molecular analysis should be routine, at least in non-SQCC, every patient with advanced NSCLC should, if at all possible, be provided with the opportunity to undergo a professional biopsy procedure. Paradoxically, although patients with advanced NSCLC usually have a higher disease bulk and more potential sites for biopsy, they still may be referred in with a sub-optimal, cytology-only fine needle aspirate (FNA) perhaps motivated by risk-avoidance. In skilled hands, and with an adequately cellular FNA, the diagnostic accuracy and value of cytology and a small biopsy are actually comparable and even complimentary (6). Importantly, both cytology and core biopsy can each be used for immunohistochemistry (IHC) and molecular testing for EGFR and KRAS, providing a cell block-sufficient sample is obtained. Nonetheless, most pathology departments prefer an adequate core biopsy both for histology (including IHC) and, if indicated, subsequent molecular testing.

It is now generally accepted that only two major types of NSCLC exist, ADC and SQCC, with other types being relatively uncommon (7). SQCC is diagnosed by the presence of keratinization and intercellular bridges, and the absence of features typical of ADC (intracellular mucin and gland formation). If this distinction cannot be rendered by conventional stains, IHC is usually adequate and highly valuable (8). Several investigations have confirmed that cytokeratin 5/6 (CK5/6) and p63 are most useful for SQCC, with a recent study showing for CK 5/6, a sensitivity of 94%, specificity of 97%, PVP of 97%, and PVN of 96%. These values for p63 were 93% (sensitivity), 87% (specificity), 96% (PVP), and 94% (PVN) (9).

Additional criteria include negativity for the ADC markers of CK7, TTF-1, and Napsin A. Although there is some variability in these results, it should be generally noted the SQCC of the lung are likely to be both CK 5/6 and p63 positive, and negative for TTF-1 or CK7. Novel immune panels continue to be developed, and which may prove superior (10).

## Molecular Pathogenesis and Targeted Therapy

The effort to elucidate the molecular abnormalities in cancer is driven not just by curiosity or technical prowess but by a deeply embedded belief system that mutations drive malignant behavior, and that a description of these “driver mutations” will inevitably lead to the design of targeted drugs that are efficacious, via inhibition of the causal chain, and non-toxic, because of the specificity of the mutation for the cancer. Although this paradigm is overly simplistic, it has proven accurate for ADC in respect to EGFR mutations, and translocation of ALK; in both cases, reliable molecular tests are available, which guide the selection of available targeted drugs, which exhibit marked if temporary activity in subgroups of ADC with these genetic alterations. Unfortunately, EGFR and ALK rearrangements have only been described in rare cases of pure SQCC (11–13), but not at a frequency that would justify testing for either mutation routinely. Furthermore, at least in the case of those rare pure SQCCs with mutated EGFR, the targeted first generation tyrosine kinase inhibitors (EGFR-TKIs) seen to be somewhat less active than in ADC with mutated EGFR (12). Consequently, unless there is pathological evidence of a mixed adeno-squamous pathology (in which case it is worth testing) (14), it is not cost-beneficial to submit SQCCs (nor, probably, “NOS – probably squamous”) cases for molecular analysis.

Likewise, KRAS mutations, which are common in (ex-) smokers with ADC, are only occasionally detected in SQCC (6.4% in the West, 1.8% in Asia) (15). KRAS in any event remains directly undruggable, but some believe that NSCLC patients with KRAS mutations may be less responsive to EGFR-TKIs (16). If true, this could explain why SQCCs do relatively well on EGFR-TKIs, despite the absence of EGRF mutations (17).

Squamous (epidermoid) lung cancer, despite the general absence of EGFR, ALK, and KRAS mutations, is genomically speaking, a highly aberrant malignancy (18). This is likely related to its close association with tobacco smoke, which contains over 5000 identified compounds, of which 73 are known carcinogens (19). These compounds form DNA adducts once metabolically activated. Unless repaired, these DNA lesions cause permanent mutations in the complementary strand due to bypass polymerases “inserting the wrong base opposite the adduct” (19). As a result of long-term exposure, thousands of mutations occur in the respiratory cells of smokers, some of which affect the function of growth regulatory genes. A variety of other processes facilitate tumorigenesis, such as inflammatory generation of reactive oxygen species and gene promoter methylation.

The Cancer Genome Atlas Research Network (CGARN) study on lung SQCC revealed many varied DNA alterations (“… a mean of 360 exonic mutations, 323 altered copy number segments and 165 genomic rearrangements per tumor”) (18). Importantly, copy number aberrations do not necessarily imply point mutations in the DNA sequence. The mean rate of exonic somatic mutations was, at 8.1 mutations per megabase, higher than any other cancer type except melanoma. TP53 mutations occurred in at least 81% of 178 samples of SQCC; TP53 is well known as the “guardian of the genome.” Other mutations occurred quite often in pathways felt to be important in the mediation of the malignant phenotype, e.g., oxidative stress (KEAP1, 12%, CUL3, 7%, and NFE 2L2, 19%); squamous differentiation (SOX2, 21%; TP63, 16%; NOTCH 1 and 2, 8% and 5%, respectively; ASCL4, 3%; and FOXP 1, 4%). mRNA expression profiling revealed overexpression of SOX2, TP63, and P1K3CA, corresponding to the known 3q26 chromosomal amplicon. The p40 version of p63 may act as an oncogene, expressed by 89% of tumors; RB1 and PTEN (loss-of-function) were also frequent. Amplification or alteration of FGFR 1, 2, and 3 were seen in 7, 3, and 2%, respectively, as well as others involved in PI3K/RTK/RAS signaling (EGFR 9%; ERB B2 4%; ERB B3 2%; PTEN 15%; PIK3CA 16%; AKT3 16%; STK 11, 2%; TSC 1 and 2, 3% each; KRAS 3%; HRAS 3%; NF1 11%; RASA 1 4%; BRAF, 4%).

CDKN2A, a “known tumor suppressor gene,” encodes INK 4A/p16 and ARF/p14, which control the cell cycle. This gene is inactivated in 77% of SQCCs (by a variety of mechanisms), often by epigenetic silencing (21%) or homozygous deletions (19%). On the other hand, about 30% overexpress both p16 and p14; often with mutation (see Tables 1 and 2).

**Table 1:** **Selected genomic alterations in SqCC**.

	Gene	Mutation rate	Normal function	Consequence of alteration	Comment
(a)	KEAP1	12%	Oxidative stress response	Loss-of-function	
(a)	NFE2L2	19%	Oxidative stress response	Activation	
(a)	CUL3	7%	Oxidative stress response	Loss–of-function	
(b)	SOX2	Zero	Squamous differentiation	Activation	Amplified in 21%
(b)	NOTCH1	8%	Squamous differentiation	Mostly loss-of-function	Mutually exclusive with TP63 or SOX2 alterations
(b)	TP63 (p40 isoform)	16%	Squamous differentiation	Activation, oncogene	
(c)	TP53	≥81%	Genomic integrity, apoptosis	Loss-of-function	Disabled in ~90% SqCC
(d)	CDKN2A	15%	Cell cycle control	Loss-of-function	Inactivated in 72% by several mechanisms
(d)	RB1	7%	Cell cycle control	Loss-of-function	Mutually exclusive with CDKN2A alterations
(e)	NF1	11%	RAS inhibitor	Loss-of-function	
(e)	BRAF	4%	Signal transduction	Activation	
(e)	RASA1	4%	RAS inhibitor	Loss-of-function	
(e)	KRAS	<1%	Signal transduction	Activation	Very uncommon in SqCC
(f)	HLA-A	3%	Antigen display	Loss-of-function	May permit avoidance of immune destruction
(g)	PTEN	8%	PI3K/Akt pathway inhibitor	Loss-of-function	
(g)	PIK3CA	16%	PI3K/Akt pathway growth and survival	Activation	AKT3 also activated in 16%
(h)	FGFR1	Few	RTK growth/survival	Activation	Amplified in 21%
(h)	EGFR	±1% L861Q mutation rate	RTK in growth/survival growth function	Activation	Amplified in 9%, rarely mutated
(i)	MLL2	20%	Chromatin regulation	?	

**Table 2:** **Alterations in major pathways in SqCC**.

Gene	Direction of dysregulation	Incidence	Normal function	Current targetability
EGFR	↑	$\begin{array}{l} 9\%\\ 4\%\\ 2\%\\ 7\%\\ 3\%\\ 2\% \end{array}\}$	
ERBB2	↑		Receptor tyrosine kinase: 26%	(Potentially targetable)
ERBB3	↑			
FGFR1	↑			
FGFR2	↑			
FGFR3	↑			
KRAS	↑	$\begin{array}{l} 3\%\\ 3\%\\ <1\%\\ 4\%\\ 11\% \end{array}\}$	
HRAS	↑		RAS/RAF: 24%	(Potentially targetable)
NRAS	↑			
RASA1	↓			
NF1	↑			
PIK3CA	↑	$\begin{array}{l} 16\%\\ 4\%\\ 16\%\\ 15\% \end{array}\}$	
AKT2	↑		PI3K/AKT: 47%	(Potentially targetable)
AKT3	↑			
PTEN	↓			
CDKN_2_A methylation	↓	$\begin{array}{l} 21\%\\ 18\%\\ 4\%\\ 29\% \end{array}\}$	
CDKN_2_A mutation	↓		CDKN_2_A: 72%	(Not currently targetable)
CDKN_2_A Exon skip.	↓			
CDKN_2_A Hom del	↓			

These changes were seen against a background of a high mutational load in apparently non-contributory genes.

The authors contemplated the totality of this picture in terms of potential therapeutic targets. They felt the location of mutations in key cancer genes, such as a variety of tyrosine kinases, serine/threonine kinases, PI3K regions, proteases, and G-protein coupled receptors, suggested potential therapeutic targets. Unfortunately, however, many of the mutations were inactivations of tumor suppressor genes, which are currently not directly druggable. FGFR alterations, however, are among the most promising, and have recently been extensively reviewed in Ref. (20).

FGFR fibroblast growth factor receptor, is a family of 4 kinase receptors (FGFR 1–4) spanning the cell membrane and involved in signal transduction via downstream RAS/MAPK and PI3K/AKT pathways. In normal physiology, FGFR signaling is involved in angiogenesis and organogenesis. In lung cancer, serum FGF levels are known to be elevated. Amplification of FGFR 1 may be characteristic of SQCC. The prognostic and predictive significance of these pathway alterations remains under investigation, but the bulk of evidence seems to suggest over-activity correlates with a poorer outcome.

The preclinical data have been sufficiently compelling to warrant the design and trialing of small molecules with FGFR inhibitory activity, including cediranib, nintedanib, pazopanib, and ponatinib. Nintedanib (an inhibitor of VEGFR 1–3, FGFR 1–4, PDGFR, FLT-3, and src), is most advanced, with a positive randomized trial (LUME – Lung 1) in second line (docetaxel ± nintedanib) (21). About 42% of the patients had SQCC; in these, the PFS HR 0.77 was significant (*p* = 0.02), as it was in the ADC subgroup (HR 0.77 *p* = 0.0193). Disease control was superior in the SQCC patients (49.3% vs. 35.5%, *p* < 0.0001). However, the effect on overall survival (OS) (in an exploratory analysis) seemed better in ADC than SQCC. Nintedanib appears tolerable, with GI and liver function abnormalities being most prominent. An excess of 12.3% of the patients required a dose reduction over placebo. LUME-Lung 3 is a first-line phase I/II trial of nintedanib with cisplatin/gemcitabine (NCT01346540).

Ponatinib is undergoing a phase II in SQCC as monotherapy after prior treatment, with FGFR amplification as an eligibility requirement (NCT01761747). A randomized trial of first-line carboplatin/paclitaxel ± cediranib was halted early for futility (22). Cediranib, however, has much less FGFR inhibitory activity than VEGFR blockade.

Pazopanib, another broad-spectrum kinase inhibitor whose actions include FGFR blockade as ancillary to VFGFR inhibition (23), is in a range of trials in A-NSCLC as monotherapy maintenance after first-line chemotherapy (NCT01208064) and with a variety of other traditional drugs in phase II. Other FGFR inhibitors include AZD4547 and BGJ398 and both are in phase I and/or phase II. FP-1039 is a FGF ligand trap also in phase I.

A series from the Massachusetts General Hospital found 16% of 226 SQCCs exhibited FGFR 1 amplifications, but these were not associated with any particular clinical features, suggesting that molecular testing would be required as a biomarker. Furthermore, the amplification was focal (24).

Looking more broadly at the pathway level, the CGARN found, in their 178 SQCC, 69% had an alteration in one of the PI3K/AKT, RTK, or RAS pathways, when considering either mutation in the DNA or amplification. For instance, 26% had either EGFR amplification, an activating BRAF mutation or FGFR 1 amplification, any of which might be targetable by an inhibitory drug. As noted, in the Canadian BR21 trial of erlotinib (an EGFR-TKI) vs. placebo, the HR in favor of drug was 0.66 in SQCC with a smoking history, almost certainly due to the driver activity of wild-type EGFR (which is quite often overexpressed at the protein level) (25).

Finally, a significant proportion of specimens contained inactivating mutations in the HLA-A gene; this might convey resistance to emerging immunomodulatory regimens. It should also be noted that human papilloma virus, a known carcinogen in the urogenital tract, and in upper respiratory epithelium, has been ruled out as instrumental in lung cancer (26).

Targetable genetic alterations in SQCC were also reviewed recently by Heist et al. from the Massachusetts General Hospital (27), emphasizing that inactivated tumor suppressor genes (tsgs) can only, if at all, be indirectly targeted. They focus on commonly mutated genes (TP53, GRM8, BAI3, ERBB4, RUNX1T1, KEAP1, FBXW7, and KRAS) while noting no currently available agents directed at these mutations. However, they do highlight genomic amplifications and areas of overexpression, which, whether mutated or not, are likely implicated as “drivers,” including SOX2 (amplified in 20% of SQCC; and a key stem cell regulator; no drugs in development); PIK3CA, affecting cell survival and proliferation (copy no. gains in >20% SQCCs, mutated in 6.5%) for which several drugs are in development (Table 3), especially buparlisib, which is in phase II and GDC-0032; and FGFR 1 (discussed above, and mediating growth, survival, and angiogenesis, for which several drugs are in development e.g., AZ4547 a specific FGFR 1–3 blocker). Although available drugs (apart from AZ4547) inhibit more than just the FGFR system, preclinical work confirms that pure inhibitors of FGFR 1 will inhibit growth of FGFR1 amplified tumor cell lines; IGF1R (insulin-like growth factor receptor) is overexpressed in some SQCCs, acting via the canonical growth and survival pathways, but promising phase II results in SQCC lung were not replicated in two phase III trials of figitumumab (a monoclonal antibody against IGF1R).

**Table 3:** **Molecularly targeted drugs under investigation in Sqcc^a^**.

FGFR inhibitors	Cediranib; nintedanib; pazopanib; ponatinib; AZD4547; BGJ398; FP-1039
EGFR inhibitor	Afatinib; necitumumab
PIK3CA	Buparlisib; GDC-0032
CDK 4/6	Palbociclib
VEGF-R	Ramucirumab, motesanib
PARP	Veliparib
Clusterin	Custirsen

*^a^Some of these agents have multiple other mechanisms of action in addition*.

Other promising targets include EphA2 (overexpression increases invasiveness, and dasatinib is an available inhibitor); MET (amplified in about 6% of SQCCs, and mediating proliferation and invasion; multiple small molecules and antibodies are in development, but recently a large phase III trial of erlotinib ± onartuzumab failed, despite a prior highly promising randomized phase II in MET overexpressors (28). A platinum/paclitaxel ± onartuzumab randomized phase II is pending (NCT01519804). Rilotumumab, an inhibitor of the MET ligand HGF/SF, is also of interest. Other targets of promise include PDGFRA (amplified in 8–10% of SQCC; sunitinib an available inhibitor); p53/MDM2 (p53 mutations in about 65% of SQCCs, mostly loss-of-function; alternatively, MDM2 overexpression can inactivate p53, as in about 7% of SQCC; no drugs yet in development); AKT(mutated in about 5% of SQCCs; several drugs in development); DDR2 (a RTK promoting migration, proliferation, and survival, and mutated in about 4% of SQCCs; dasatinib may be active); LKB1 (a cell cycle regulator, inactivated in 5–20%; not yet drugged); PTEN (a tsg, and negative regulator of PI3K/AKT, which is then de-repressed when PTEN inactivated, very frequent in both types of NSCLC, especially SQCC; PI3K inhibitors are logical here); NRF2/KEAP1 (an oxidative stress response system; KEAP1 negatively regulates NRF2, and dysregulation of either gene is common in SQCC; no drugs in development yet).

The cyclin-dependent kinases, CDK 4 and 6 are another target of interest and the inhibitor palbociclib (PD-0332991) is in phase II in SQCC.

EGFR is commonly overexpressed (but rarely mutated) in SQCC. The overexpression may be associated with amplification or polysomy (29–31). FLEX, a first-line phase III trial of cisplatin/vinorelbine ± cetuximab, was especially positive in both types of NSCLC if, in an explanatory analysis, EGFR was overexpressed by IHC, independent of mutation status (32, 33), but this remains controversial. As noted, BR.21, a last-line study of erlotinib vs. placebo, showed a beneficial HR 0.67 for SQCC. A 545 patient, first-line phase III trial of cisplatin/gemcitabine ± necitumumab, a novel, fully human lgG1 monoclonal antibody, has just been reported as positive for OS (median survival 11.5 vs. 9.9 months, HR 0.84, *p* = 0.012). One- and 2-year survival also favored the necitumumab arm (34). Patients <70 years seemed to fare better, but the H-score biomarker seemed to exhibit at most a trend for more benefit at higher levels of EGRF expression. Another (phase II) RCT is testing carboplatin/paclitaxel ± necitumumab in first-line SQCC (NCT0176939). Some ±5% patients with SQCC exhibit a truncated form of EGFR known as EGFR-vIII, which is transforming, but which lacks an extra-cellular domain, and may be less amenable to inhibition by antibody. Lux-Lung 8 is an ongoing phase III study of a novel, pan-HER, irreversible inhibitor afatinib, vs. erlotinib, in last-line SQCC, and initial reports indicate a modest superiority over erlotinib (35). It remains an open question as to whether EGFR by IHC or gene copy number, can suffice as a biomarker for benefit from anti-EGFR agents in SQCC.

The VEGF/VEGFR angiogenesis pathway is clearly implicated in SQCC progression (36). Bevacizumab, a VEGF-sequestration antibody, caused an unacceptable rate of fatal hemoptysis in SQCC (37). Whether this relates to the tendency of SQCC to have cavitating primaries close to major airways, or some more complex association with squamous histology, is still unresolved; however, bevacizumab was only approved for use in non-SQCCs, for safety reasons. Bevacizumab may well be active in SQCCs and may be safer in patients with excised primaries; this is not known and would be off-label.

Ramucirumab, a fully human lgG1 monoclonal antibody to VEGRF-2, was recently the subject of a randomized phase III trial (REVEL: docetaxel ± ramucirumab) in second-line NSCLC including SQCC. It was positive for OS; REVEL included 328 SQCC patients, who experienced a modest OS benefit (9.5 vs. 8.2 months, HR 0.88) in a subgroup analysis (38). Motesanib, a small molecule VEGF-R inhibitor, seemed ineffective and prohibitively toxic in a recent randomized trial (39).

It is noteworthy that the successful RCTs in SQCC involve agents not directed at mutated proteins, but at normal components of upregulated pathways in which the specificity arises from contextual, causal dependence rather than biomolecular structural differences. Furthermore, as is usual, the benefits, albeit welcome, appear to be temporary. With the exception of chronic myeloid leukemia and bcr-abl inhibitors, targeted drugs based on the molecular causality principle have uniformly provided only temporary benefit.

## Recognition-Based Targeting

Interference in the causal chain mediating malignancy is not the only way to target cancer; another way is based on the principle of recognition. The immune system exploits this to protect us very effectively against foreign organisms, and also, cellular transformation; its direct targets are surface-based biomolecular differences (“markers”), irrespective of whether they are causally important drivers or not. Until recently, it was quite erroneously believed that most cancers were not “antigenic enough” to evolve an effective immune response (IR); however, it is now appreciated that the problem is more that the local milieu within the tumor environment is immunosuppressive, partly because of the manipulation of the immune system by negative regulators expressed on the tumor cells, and (still under-appreciated) the highly proteolytic and extremely acidotic extra-cellular milieu in tumors (40), which is likely to damage the three-dimensional structure of extra-cellular recognition peptides on which the IR is utterly dependent.

Squamous (epidermoid) lung cancer cells, by virtue of the very high mutation burden, are likely to express altered proteins as efficient neoantigens in the context of HLA. (The latter, as noted, may be mutated, potentially compromising antigen presentation in SQCC.) New immunomodulatory agents, also known as “immune checkpoint inhibitors,” which have proven effective in both forms of lung cancer (as well as melanoma), derive from an understanding of the “immunological synapse” (41), a complex network of positive and negative regulatory interactions that occur among the tumor cells, the dendritic antigen-presenting cells and the T lymphocytes, and which strongly influence whether these effector (cytotoxic) T-cells are activated or not.

So far, useful therapies have been developed to block CTLA4 (an early negative regulator on T-cells, active in draining lymph nodes), with a monoclonal antibody, and the PD-L1/PD1 interaction (with monoclonal antibodies directed against either the PD-1 negative regulator on the T lymphocytes, or the PD-L1 ligand on the tumor cell, including SQCC cells, or dendritic cell). This system acts later in the “cancer-immunity cycle,” (42), in the actual tumor milieu. Monoclonal antibodies against all three targets have shown surprising activity in lung cancer, and are in accelerated development; side effects have generally been tolerable, with a range of auto-immune effects, more associated with the anti-CTLA4 monoclonal antibody ipilimumab, and rare but potentially serious pneumonitis with the prominent anti PD-1 agent nivolumab.

The CA184-041 study tested two regimens of combination ipilimumab with chemotherapy (concurrent or phased) vs. chemotherapy only; the phased (but not the concurrent) PFS was significantly superior to the chemotherapy only arm. The SQCC patients seemed to benefit more (HR 0.48 for OS) (43). NCT01285609, an ongoing phase III, tests the combination vs. chemotherapy in first-line SQCC, using the apparently superior phased regimen.

The anti-PD1 agent nivolumab achieved a 6/18 ORR (SQCC) in a phase I study; despite heavy pre-treatment, OS at 2 years was 24% across all 129 NSCLC patients (44). NCT01721759 is an ongoing phase II in SQCC (third line). A phase III in SQCC (nivolumab vs. docetaxel) is underway (NCT01642004), as are phase I trials in A-NSCLC with the various platinum doublets, and also in the highly promising combination with ipilimumab. The role of tumor cell expression of PD-L1 as a biomarker is being investigated in these studies. MK-3475 in another anti-PD1 monoclonal antibody with a 2/6 ORR in SQCC, being moved into phase II/III (NCT01905657), and another pending phase III (NCT0214738), also focused on PD-L1 expressing tumors.

Several anti-PD-L1 antibodies have also shown some activity against SQCC such as BMS-936559 (45). MPDL3280A is another anti-PD-L1 monoclonal with early demonstrated activity in SQCC (3/20), especially associated with IHC positivity of PD-L1 (46). NCT01903993 will compare MPDL3280A with docetaxel in 2L, followed by the phase III (NCT02008227). MEDI4736 is another anti PD-L1 agent entering phase II in advanced NSCLC (ATLANTIC NCT02087423) and phase III in stage III (PACIFIC), including SQCC.

Readers are referred to two excellent overviews for further details (47, 48), and to Table 4.

**Table 4:** **Checkpoint inhibitors under evaluation in advanced SqCC (selected studies)**.

Clinical Trials.gove Identifier, agent, trial name	Target	Phase	Line	Design	Status
NCT01285609	CTLA-4	III	1st	Carboplatin/paclitaxel ± Ipilimumab	Recruiting
Ipilimumab					
NCT01721759	PD-1	II	3rd	Single agent nivolumab	Active, not recruiting
Nivolumab					
Checkmate 063					
NCT01642004	PD-1	III	>1st	Nivolumab vs. docetaxel	Active, not recruiting
Nivolumab					
Checkmate 017					
NCT02041533	PD-1	III	1st	Nivolumab vs. investigator’s choice chemotherapy	Recruiting
Nivolumab					
Checkmate 026					
NCT01454102	PD-1	I	Multiple	Nivolumab with various platinum doublets and/or biologicals/targeted agents including ipilimumab, erlotinib	Recruiting
Nivolumab					
Checkmate 012					
NCT01295827	PD-1	I/II	≥1st	Low and high doses, q2 and 3 week schedule	Active, not recruiting
Pembrolizumab					
KEYNOTE 001					
NCT02220894	PD-1	III	1st	Pembrolizumab vs. carboplatin/paclitaxel (or pemetrexed)	Not yet open
Pembrolizumab					
KEYNOTE 042					
NCT02039674	PD-1	I/II	≥1st	Pembrolizumab with various platinum doublets and/or biologicals/targeted agents	Recruiting
Pembrolizumab					
KEYNOTE 021					
NCT02007070	PD-1	II	2nd	Single agent	Recruiting
Pembrolizumab									PTO
KEYNOTE 025					
NCT01905657	PD-1	II/III	≥2nd	2 doses of Pembrolizumab vs. docetaxel	Recruiting
Pembrolizumab	
KEYNOTE 010					
NCT0214738	PD-1	III	1st	Pembrolizumab vs. platinum doublet	Not yet recruiting
Pembrolizumab					
KEYNOTE 024					
NCT01846416	PD-L1	II	≥1st	Single agent	Active, not recruiting
MPDL3280A					
FIR					
NCT02031458	PD-L1	II	≥1st	Single agent	Recruiting
MPDL3280A					
BIRCH					
NCT01903993	PD-L1	Random II	2nd	MPDL3280A vs. docetaxel	Active, not recruiting
MPDL3280A					
POPLAR					
NCT02008227	PD-L1	III	2nd	MPDL3280A vs. docetaxel	Recruiting
MPDL3280A					
OAK					
NCT02087423	PD-L1	II	3rd	Single agent	Recruiting
MEDI4736					
NCT02000947	PD-L1	I	≥1st	MEDI4736 with Tremelimumab	Recruiting
MEDI4736					
NCT02154490	PD-L1	II/III	2nd	Multi-arm master protocol vs. docetaxel for various targeted agents including MEDI4726	Recruiting
MEDI4736					
LUNG-MAP					
NCT02087423	PD-L1	II	3rd	Single agent	Recruiting
MEDI4736					
ATLANTIC					

*Ref for this table: Clinical Trials.gov accessed 31 August 2014*.

## Bone Metastases

Bone metastases may occur in any type of lung cancer, and two categories of drugs have shown efficacy in reducing skeletal-related events (SREs). The bisphosphonate zoledronic acid (49–51) is well studied, and patients with elevated osteoclast marker (N-telopeptide of type 1 collagen) appear to experience an OS benefit (52). Denosumab, a monoclonal antibody against RANK-ligand, an osteoclast activator, led to an OS benefit compared with zoledronic acid, in a large NSCLC subgroup analysis (51) (HR 0.78, *p* = 0.01) in which the biggest benefit was experienced by SQCC (HR 0.68 *p* = 0.035), and also which was associated with a lower incidence of SREs than zoledronic acid (53).

## Chemotherapy

No particular third-generation platinum doublet stands out as clearly superior in advanced SQCC, and the decision should be made based on toxicity avoidance (54). In a subgroup analysis of ECOG1594, a large four-armed phase III of taxane and gemcitabine platinum doublets, cisplatin/gemcitabine had the best PFS (4.3 months) and OS (9.4 months) in SQCC, but the differences were not statistically significant. Pemetrexed, however, whether as a single agent or in combination with cisplatin, is inferior and contra-indicated in SQCC, despite its superiority in ADC and large cell (55, 56). Pemetrexed is also ineffective in prolonging either PFS of OS in maintenance in SQCC, in contrast to non-SQCC (57). Pemetrexed may be less efficacious in SQCC because of higher thymidylate synthase levels (58, 59), although differential expression of the folate receptor alpha may also be important (60).

Nab-paclitaxel with carboplatin appears to be superior to paclitaxel/carboplatin in SQCC, with a higher ORR and less grades 3/4 neuropathy and arthralgia in SQCC (61). Neither PFS nor OS were different. The 41% ORR for the nab-paclitaxel arm is notable, as it was independently reviewed in Ref. (62).

In Japan, the LETS phase III study demonstrated the superiority of carboplatin/S-1 over carboplatin/paclitaxel in SQCC (HR 0.713; 14.0 vs. 10.6 months) (63). S1 is an oral fluoropyridine.

Further improvements in chemotherapy are unlikely to emerge without the addition of biologicals, some of which have been detailed above. One additional possibility relates to PARP inhibitors. PARP (poly ADP-ribose polymerase) is an enzyme that participates in several DNA repair pathways (64) and is believed to be important particularly in SQCC (65). An initial randomized phase II experience suggested significant benefit in SQCC with veliparib, a PARP inhibitor (PFS HR 0.50) OS HR 0.72), when added to carboplatin/paclitaxel (66) NCT01560104, a phase III trial of first-line platinum chemotherapy, ±veliparib (ABT-888), is currently underway. An earlier trial of chemotherapy ± iniparib failed, probably because iniparib might not be a sufficiently active PARP inhibitor (67).

The efficacy of chemotherapy may be also be attenuated by anti-apoptotic (pro-survival) proteins like clusterin, expressed in about 70% of NSCLC, apparently unrelated to histological subtype (68, 69). A phase I/II trial of platinum/gemcitabine with clusterin, a 2.0 generation antisense oligonucleotide, achieved an OS of 14.1 months, which was thought sufficient to justify the phase III trial now underway.

## Conclusion

The proportionate reduction in SQCC is likely to be the result of an ongoing reduction in cigarette smoking (70); however, as long as tobacco products (and, probably, marijuana) (71) are consumed, this disease will be a major public health concern. Although most of the progress in lung cancer in the last decade has occurred in ADC, a recent spate of positive trials has, at last, brightened the prospects for SQCC. OS gains have been shown for ramucirumab, necitumumab, cetuximab, denosumab, and nintedanib (to be prospectively confirmed); although these results are modest, they do provide a foundation upon which to explore novel biomarkers and potentially synergistic drug combinations. Furthermore, immunomodulators such as anti-PD-1/PD-L1 agents are unquestionably active. The rapidly expanding trove of knowledge on the volatile genome of SQCC has thrown up some further target opportunities, such as the FGFR family. This progress will likely serve to push the 1-year median OS consistently through the 1-year barrier; beyond that, further radical innovation will be necessary.

## Conflict of Interest Statement

The author declares that the research was conducted in the absence of any commercial or financial relationships that could be construed as a potential conflict of interest.
